# The correlation of Th22 and regulatory T cells with *Helicobacter pylori* infection in patients with chronic gastritis

**DOI:** 10.1002/iid3.768

**Published:** 2023-01-18

**Authors:** Biyu Yao, Xiaoyan Xu, Weijie Liu, Qin Zhang, Wei Wang, Zhiming Huang

**Affiliations:** ^1^ Department of Gastroenterology People's Hospital of Yuhuan Zhejiang Taizhou China; ^2^ Department of Gastroenterology The First Affiliated Hospital of Wenzhou Medical University Zhejiang Wenzhou China

**Keywords:** flow cytometry analysis, Foxp3, IL‐22, Pearson's correlation analysis, regulatory T cells, Th22 cells

## Abstract

**Objective:**

*Helicobacter pylori* is planted in the human stomach and is the most common cause of chronic gastritis, which produced specific local and systemic humoral immunity, while the associations of these immune responses and *H. pylori* in the development of chronic gastritis remain unclear.

**Methods:**

This study analyzed histology, the number of Th22 and regulatory T (Treg) cells, and the levels of inflammation‐ and gastritis‐related indicators between 22 *H. pylori*‐infected and 24 non‐*H. pylori*‐infected chronic gastritis patients by hematoxylin‐eosin staining, enzyme‐linked immunosorbent assay, quantitative reverse transcription PCR, and flow cytometry analysis.

**Results:**

This study found that the pathological damage degree of gastric mucosa in *H. pylori* infection patients was more serious. In the *H. pylori*‐infected patient serum, the gastrin, G‐17, interleukins (IL)‐22, transforming growth factor (TGF)‐β, tumor necrosis factor (TNF)‐α, IL‐4, and IL‐17A levels were notably raised, while the interferon (IFN)‐γ level was inhibited, and in gastric mucosa, and except IFN‐γ, the IL‐22, forkhead box P3 (Foxp3), TNF‐α, IL‐4, and IL‐17A mRNA levels were raised too. The receiver operating characteristic curve analysis indicates serum IL‐22, TGF‐β, TNF‐α, IL‐4, and IL‐17A are suitable for differential diagnosis of *H. pylori* infection. In addition, in the peripheral blood, the percentages of the IL‐22^+^ CD4^+^ and Foxp3^+^ CD4^+^ T cells were raised with *H*. *pylori* infection. The positive correlation between IL‐22 and Foxp3 mRNA levels and the degree of *H. pylori* colonization and gastric mucositis by Pearson's correlation analysis.

**Conclusions:**

Treg and Th22 cells were positively associated with the degree of *H. pylori* infection and the severity of gastritis. In summary, this study provides an experimental basis for the study of the eradication of *H. pylori* and the biological mechanism of chronic gastritis.

## Graphical abstract

This study reported that serum interleukins (IL)‐22, transforming growth factor‐β, tumor necrosis factor‐α, IL‐4, IL‐17A, and interferon‐γ levels may be suitable for the differential diagnosis of *Helicobacter pylori* infection. Furthermore, this study found a positive correlation between IL‐22 and forkhead box P3 mRNA levels and the degree of *H. pylori* colonization and gastric mucositis. Additionally, regulatory T and Th22 cells were positively associated with the degree of *H. pylori* infection and the severity of gastritis.

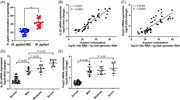

## 1 INTRODUCTION

The gram‐negative spiral bacterium, *Helicobacter pylori*, is planted in the human stomach. It is the most common cause of chronic gastritis which is an inflammatory condition of the gastric mucosa.^1^, ^2^ Chronic gastritis includes atrophic or non‐atrophic gastritis, and the complex immune response caused by *H. pylori* is the key factor in its inflammation.^3^, ^4^ In infected individuals, the activation of innate and adaptive immune systems can lead to the recruitment of a variety of inflammatory cells (including dendritic cells, macrophages, neutrophils, mast cells, T cells, and B cells) into the stomach, in which T cells are the main coordinator of immunity.^5^ The collected immune cells secrete a variety of proinflammatory cytokines, such as interleukins (IL)‐4, tumor necrosis factor (TNF)‐α, interferon (IFN)‐γ, and IL‐22, which lead to chronic inflammation unique to *H. pylori* infection. At present, it is clear that *H. pylori* infection can produce specific local and systemic humoral immunity,^6^ however, the role of these immune responses in the development of chronic gastritis remains unclear.

Th22 cells with C‐C chemokine receptor type 4, 6, and 10 molecules and secrete IL‐22, IL‐13, and TNF‐α, is a novel subset of CD4 T cells, and it can't secrete IL‐17, IL‐4, and IFN‐γ and was inducted in naive T cells by TNF‐α.^7^, ^8^ Studies have found different roles for Th22 in different experimental models, for example, in the in vitro injury model, Th22 supernatant enhanced the healing function of keratinocytes, and it's completely dependent on IL‐22.^9^ However, Yuan's team studied patients and mice with *H. pylori* infection and found that Th22 cell polarization was related to increased inflammation within the gastric mucosa, and the secretion of IL‐22 promoted the development of inflammation.^10^ In addition, regulatory T (Treg) cells have been proven as having a major regulatory role in the adaptive immune response and are involved in *H. pylori*‐related inflammatory reactions and maintenance of bacterial persistence by immunologic tolerance.^11^ In C57BL/6 mice, *H. pylori* caused the gastric forkhead box P3 (Foxp3^+^) T‐cell response, and depletion of these T cells could lead to increased gastric inflammation.^12^ While a study reported that *H. pylori* promoted the differentiation of Treg cells to maintain infection.^13^ Especially, scientists found that stimulating CD25^−^ T cells in rag^−/−^ mice without CD25^+^ T cells would promote *H. pylori* clearance, and in the *H. pylori* eradication patients, the proportion of CD25^+^ T cells in CD4^+^ T cells in gastric mucosa decreased, which means CD25^+^ cells induce anergy in CD25^−^ cells in response to *H. pylori* infection.^14^, ^15^ Treg cells may play a role in *H. pylori* stimulating Th22 cells to produce IL‐22 inducing CG. Therefore, we explore the correlation between Th22 and Treg cells in patients with chronic gastritis infected by *H. pylori*, provide an experimental basis for exploring the immune mechanism against *H. pylori*, and provide a direction for the eradication of *H. pylori* in chronic gastritis.

## 2 MATERIALS AND METHODS

### 2.1 Patients

From March 2020 to 2021, the 46 chronic gastritis patients who received diagnosed according to the results of the gastroduodenoscopy and histology at our hospital were enrolled. Diagnostic criteria were referred to previous studies^16^ and the diagnosis was performed by specialist gastrointestinal disease pathologists. Among them, the 24 chronic gastritis patients with negative results from the rapid urease test and 13C urea breathing test were classified into the *H*. *pylori*‐NC group. And the 22 patients with positive results of the rapid urease test or 13C urea breathing test were classified into the *H. pylori* group. These patients had no previous history of *H. pylori* eradication and gastric surgery or intestinal diseases without other digestive tract diseases, such as digestive tract tumors and ulcers; patients with cardiovascular and cerebrovascular diseases, liver, kidney, and blood system diseases should be excluded, and they have not taken antacids or gastric mucosal protectants in the past 2 weeks, or antibiotics in the past month; lactating and pregnant women or patients with mental disorders and serious mental disorders are also excluded. All of them were informed about this study and gave written informed consent before the start of the experiment. Basic information about the included individuals, including gender, age, duration of illness, smoking history, and drinking history, was collected, and statistically analyzed. The study protocol was approved by the Ethics Committee of the People's Hospital of Yuhuan (Zhejiang, China).

### 2.2 Specimens

On the next day of patient admission, 6 ml of venous blood from fasting patients were collected in the morning in tubes containing sodium citrate anticoagulant solution and stored for flow cytometry assay. The serum was collected after centrifugation and stored at −80°C. Also, some of the biopsy samples taken from the gastric mucosa of each patient were stored at −80°C, and the others were fixed with 10% formaldehyde and embedded in paraffin for subsequent experiments.

### 2.3 Hematoxylin‐eosin staining (H&E)

The 4 μm paraffin section of gastric mucosa was detected by an H&E kit to evaluate the gastric mucosa morphology.^17^ And the gland morphology, density, mucosal thickness, and inflammatory cell infiltration degree of gastric mucosa were observed. Evaluation of histology of gastric mucosal was according to the degree of inflammatory cell infiltration (score: 0−3), the extent of mucosa thinning compared to the normal gastric mucosa (score: 0−3), the degree of derangement in shape and arrangement of glands (score: 0−3), and the density of glands compared with normal gastric mucosa (score: 0−3), and the patients were divided into mild, moderate, and severe gastritis by semiquantitative scoring of gastric mucosa tissue sections.^18^

### 2.4 Enzyme‐linked immunosorbent assay (ELISA)

According to the manufacturer's instructions, the ELISA kits of gastrin (MM‐2499H2; Meimian), Pepsinogen I (PGI; MM‐2267H2; Meimian), Pepsinogen II (PGII; MM‐0105H2; Meimian), gastrin 17 (G‐17; ml037633; mlbio), and inflammation‐associated cytokines of IL‐4, 17A, 22 (MM‐0165M1; MM‐0170M1; MM‐1656H2; Meimian), transforming growth factor β (TGF‐β; MM‐5093302; Meimian), TNF‐α (ml077385; mlbio), and IFN‐γ (ml077386; mlbio) were used to test those cytokines' level in serum.

### 2.5 Quantitative reverse transcription PCR (qRT‐PCR)

After lysis, centrifugation, and pelleting, the RNA from gastric mucosa tissue was dissolved in 40 μl DEPC water and stored in a −80°C refrigerator for standby.^19^ The reverse transcription process was performed by HiFiScript cDNA Synthesis Kit (CW2569; cwbio). Finally, according to the reagent instructions, the samples were mixed with the SYBR Premix Ex TaqII (RR820A; Takara) kit, and the qRT‐PCR was carried out by the qRT‐PCR instrument (CFX Connect; BIO‐RAD). The data were processed by the relative quantitative method ($2^{‐\Delta \Delta C_{t}}$). Primer information was shown in Table 1. And the amount of *H. pylori* colonization is the bacterial content (copies/μl) ratio to the concentration of the sample (ng/μl).

**Table 1:** Primer sequence information

Gene	Primer
Forward primer	5′‐TTTGTTAGAGAAGATAATGACGGTATCTAAC‐3′
Reverse primer	5′‐CATAGGATTTCACACCTGACTGACTATC ‐3′
TaqMan probe	5′‐FAM‐ CGTGCCAGCAGCCGCGGTTAMRA‐3′

### 2.6 Flow cytometry assay

Fresh anticoagulation‐treated peripheral blood was used for flow cytometry assay.^20^, ^21^ For IL22^+^CD4^+^ cells, in brief, peripheral blood mononuclear cells were separated by the way of Ficoll‐Paque and inoculated into RPMI‐1640 cell culture with 10% fetal bovine serum (11011‐B611; SIJIQING) and 1% penicillin‐streptomycin solution (SV30010; HyClone), and it cultured in the 24‐well plates at 37°C in 5% CO_2_. Cells were stained with anti‐human CD4 FITC (566320) for 15 min. Then the cells were incubated with GolgiStop protein transport inhibitor (554724; BD Pharmingen), phorbol 12‐myristate 13‐acetate (B50767; Yuanye), and calcium ionomycin (SQ23377; MCE) for 5 h. Subsequently, cells were performed with fixation/permeabilization working solution and permeabilization buffer. After the sediment was washed, the sediment was taken and prepared into a 10^6^/ml suspension. The suspension was treated with anti‐human IL‐22 (567563) avoiding light for 50 min at 4°C. After cleaning, suspending, and sieving again, samples were carried out by BD Accuri™ C6 flow cytometer (BD). A total of 6 × 10^4^ events were acquired for each sample via flow cytometry. Additionally, for Treg cells, the isolated cells were stained with anti‐human CD4 and anti‐human CD25 (555432) for 30 min. Subsequently, cells were fixed and permeabilized with fixation/permeabilization working solution and permeabilization buffer for 20 min at RT in the dark. Finally, stained cells with anti‐human Foxp3 antibody (561493). And all antibodies were bought from Becton, Dickinson and Company.

### 2.7 Statistical analysis

If the measurement data of multiple groups conform to the normal distribution and the variance is homogeneous, the one‐way‐ANOVA following the Tukey test is used, and two‐group data were analyzed by *t*‐test. If it does not conform to the normal distribution, the Kruskal−Wallis *H* test shall be used. The receiver operating characteristic (ROC) curve analysis of was used to determine the discriminative power of serum markers for *H. pylori* infection. The Pearson's correlation analysis was used to analyze the correlation between the IL‐22 and Foxp3 mRNA levels and the degrees of *H. pylori* colonization and gastric mucositis. And partial correlation analysis was used to analyze the correlation after controlling variables. All data were analyzed by SPSS 25.0. and expressed as mean ± standard deviation, *p* < .05 means the difference was statistically significant.

## 3 RESULTS

### 3.1 Baseline patient characteristics

The baseline patient characteristics of the two groups have been collected and analyzed, and the results are shown in Table 2. A total of 46 patients were included in the study. There were 13 females and 11 males in the *H. pylori*‐NC group with a median age of 33.67 ± 3.33 years (range 28−38 years), and 12 females and 10 males in the *H. pylori* group with a median age of 33.32 ± 3.30 years (range 28−39 years). The common data of patients in the two groups were not statistically significant (*p* > .05) (Table 2). Additionally, the sample size and experimental process are shown in the Supporting Information: Figure 1.

**Table 2:** Baseline patient characteristics

Patient information	*Helicobacter pylori*‐NC	*H. pylori*	*p* Value
	(*n* = 24)	(*n* = 22)	
Sexual distinction			.979
Male	11	10	
Female	13	12	
Age	33.67 ± 3.33	33.32 ± 3.30	.724
Duration of illness (month)	31.54 ± 15.10	34.59 ± 17.72	.532
Smoke	3	3	.909
Drink wine	5	4	.821

### 3.2 The levels of gastritis‐ and inflammation‐associated cytokines in the serum of patients with chronic gastritis correlate with *H. pylori* infection

**Figure 1:**
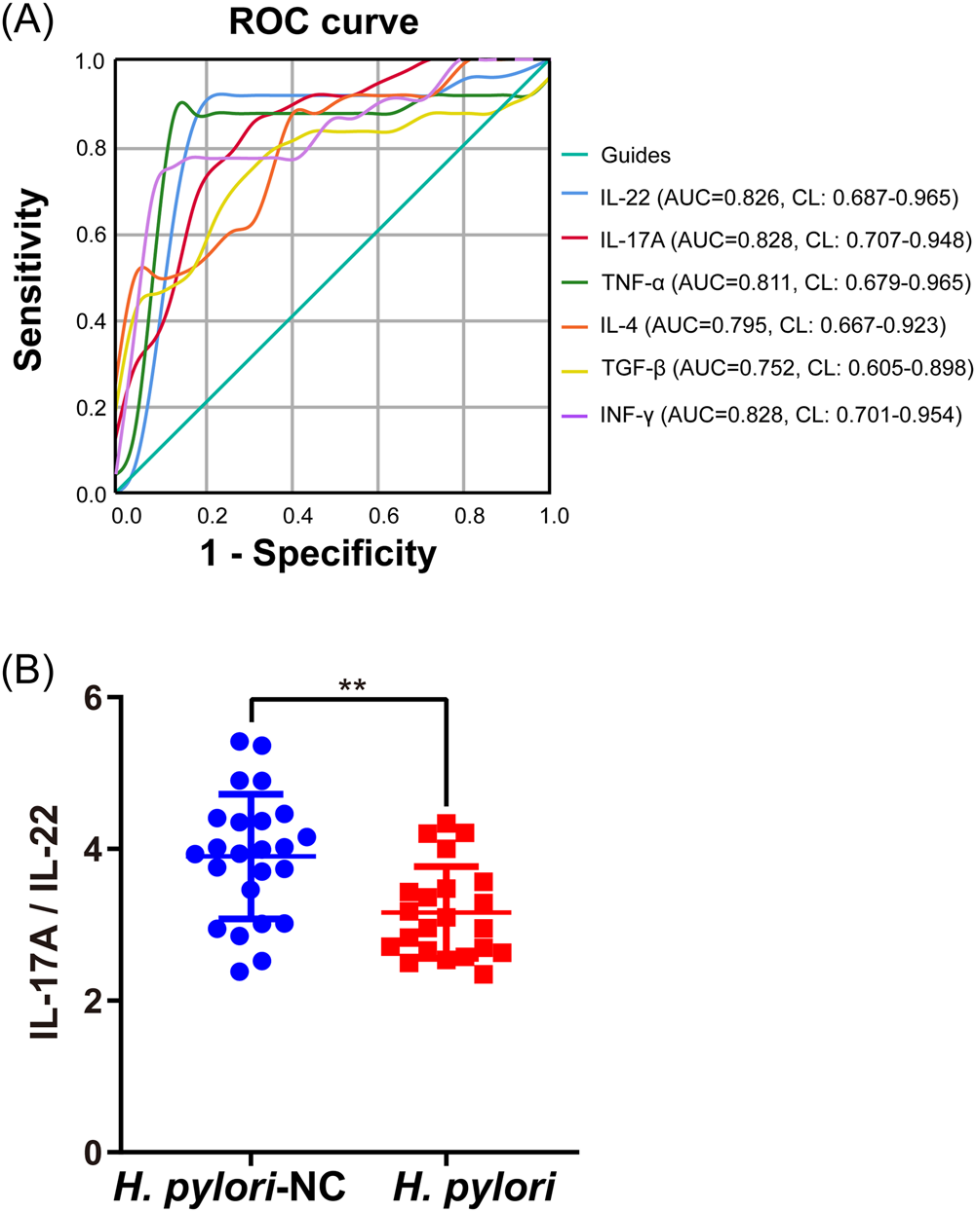
The results of ROC curve analysis and IL‐17A/IL‐22 in the gastric mucosa of patients with chronic gastritis. (A) The ROC curve analysis of IL‐22, TGF‐β, TNF‐α, IL‐4, IL‐17A, and IFN‐γ was performed by SPSS 25. The AUC was IL‐22 (0.826), IL‐7A (0.828), TNF‐α (0.811), IL‐4 (0.795), TGF‐β (0.754), and IFN‐γ (0.828). (B) The ratio of IL‐17A level to IL‐22 level in the serum that measured by ELISA. AUC, area under the ROC curve; ELISA, enzyme‐linked immunosorbent assay; IFN, interferon; ROC, receiver operating characteristic; TNF, tumor necrosis factor.

The levels of gastrin, PGI, PGII, G17, IL‐22, TGF‐β, TNF‐α, IFN‐γ, IL‐4, and IL‐17A in blood were measured by ELISA (Table 3). In the *H. pylori* group compared to the *H. pylori*‐NC group, the levels of gastrin, G17, IL‐22, TGF‐β, TNF‐α, IL‐4, and IL‐17A were significantly raised (*p* < .01). Especially, the IFN‐γ level was inhibited markedly (*p* < .01). Except for these, the degrees of PGI and PGII were not statistically significant (*p* > .05). Subsequently, the ROC curve analysis of IL‐22, TGF‐β, TNF‐α, IL‐4, IL‐17A, and IFN‐γ was performed by SPSS 25.0. The area under the ROC curve (AUC) was IL‐22 (0.826), IL‐7A (0.828), TNF‐α (0.811), IL4 (0.795), TGF‐β (0.752), and IFN‐γ (0.828) (Figure 1A). And the ratio of IL‐17A to IL‐22 was significantly higher in *H. pylori‐*NC group than it was in the *H. pylori* group (Figure 1B).

**Table 3:** The levels of gastritis‐ and inflammation‐associated cytokines in the serum

Group	*Helicobacter pylori*‐NC	*H. pylori*	*p* Value
Gastrin (mg/L)	73.97 ± 9.23	128.89 ± 16.29	<.001
PGI (mg/L)	83.48 ± 12.05	86.49 ± 13.78	.443
PGII (mg/L)	7.52 ± 0.88	7.95 ± 1.24	.347
G17 (mg/L)	1.59 ± 0.67	2.93 ± 1.05	<.001
IL‐22 (pg/ml)	23.99 ± 3.17	46.61 ± 6.59	<.001
TGF‐β (pg/ml)	257.48 ± 39.06	317.71 ± 49.57	.003
TNF‐α (pg/ml)	62.29 ± 8.34	97.64 ± 12.96	<.001
IFN‐γ (pg/ml)	446.27 ± 50.39	359.23 ± 35.73	<.001
IL‐4 (pg/ml)	196.09 ± 30.61	248.18 ± 36.71	<.001
IL‐17A (pg/ml)	92.18 ± 16.28	145.18 ± 21.77	<.001

Abbreviations: IFN, interferon; IL, interleukins; PGI, pepsinogen; TGF, transforming growth factor; TNF, tumor necrosis factor.

### 3.3 The effect of *H. pylori* infection on the gastric mucosa of patients with chronic gastritis

**Figure 2:**
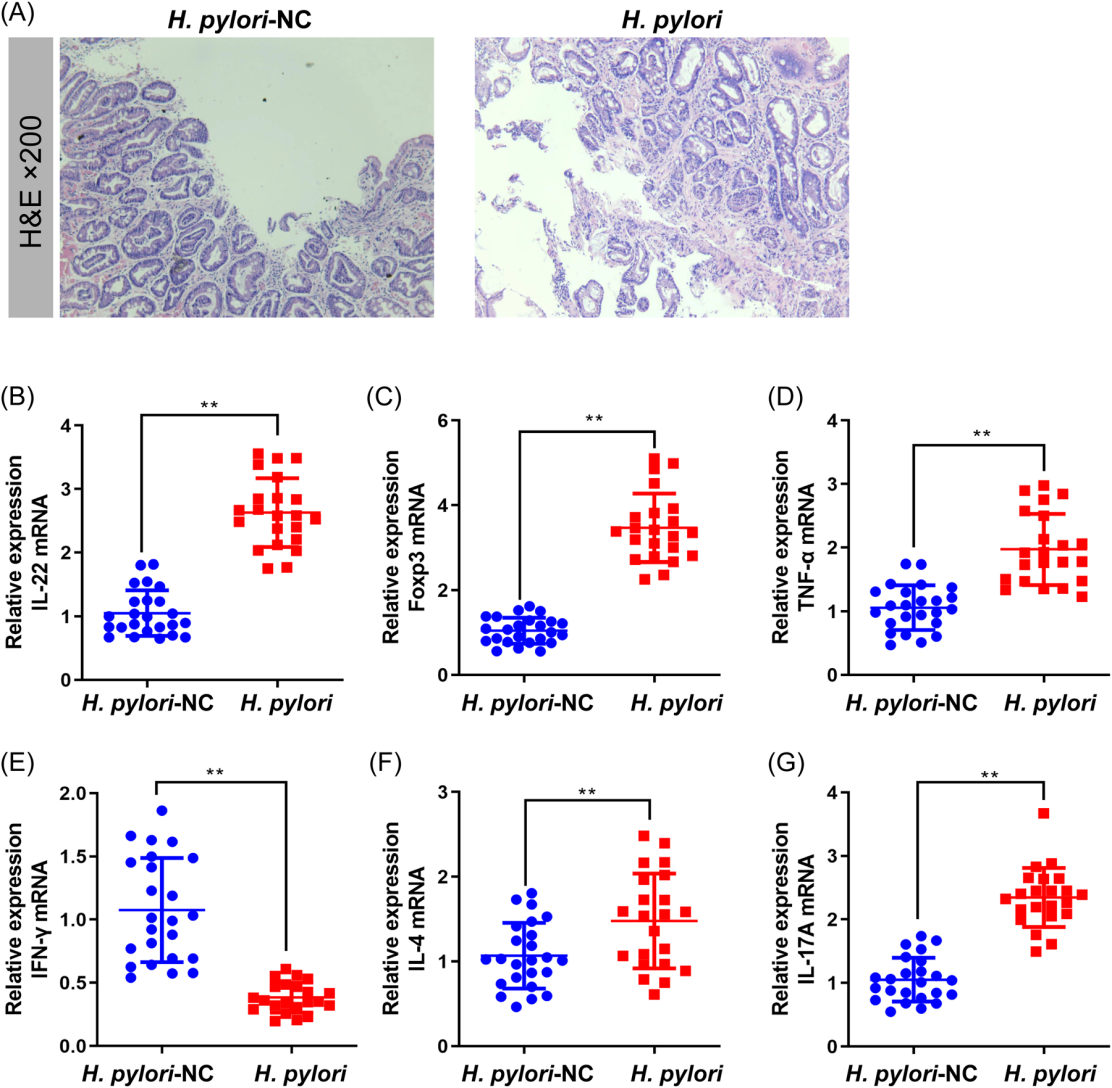
The results of H&E staining and qRT‐PCR in the gastric mucosa of patients with chronic gastritis. (A) The representative photos of H&E staining in the *H. pylori*‐NC group and the *H. pylori* group (×200). The levels of (B) IL‐22, (C) Foxp3, (D) TNF‐α, (E) IFN‐γ, (F) IL‐4, and (G) IL‐17A mRNA in the gastric mucosa of patients with chronic gastritis were measured by qRT‐PCR (mean ± SD) **p < .01 versus the *H. pylori* group. Foxp3, forkhead box P3; H&E, hematoxylin‐eosin staining; *H. pylori*, *Helicobacter pylori*; INF‐γ, interferon γ; IL, interleukin; qRT‐PCR, quantitative reverse transcription PCR; SD, standard deviation; TNF‐α, tumor necrosis factor α.

As observed by H&E staining, the glands of the gastric mucosa of *H. pylori*‐infected patients were arranged in a disorganized, less dense, and more significantly infiltrated by inflammatory cells than the control patients (Figure 2A). The mRNA levels of IL‐22, Foxp3, TNF‐α, IFN‐γ, IL‐4, and IL‐17A in the gastric mucosa were measured by qRT‐PCR (Figure 2B−G). It showed that the degrees of IL‐22, Foxp3, TNF‐α, IL‐4, and IL‐17A mRNA were raised by *H. pylori* infection, while the IFN‐γ mRNA level was downregulated markedly (*p* < .01).

### 3.4 The effect of *H. pylori* infection on the number of IL‐22^+^ CD4^+^ and Foxp3^+^ CD4^+^ T cells in peripheral blood of patients with chronic gastritis

**Figure 3:**
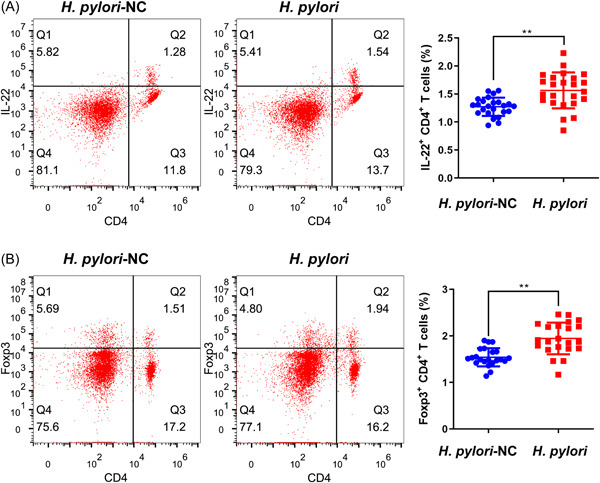
The results of the flow cytometry assay. The effect of *H. pylori* infection on the number of (A) IL‐22^+^ CD4^+^ and (B) Foxp3^+^ CD4^+^ T cells in peripheral blood of patients with chronic gastritis (mean ± SD) **p < .01 versus the *H. pylori* group. Foxp3, forkhead box P3; *H. pylori*, *Helicobacter pylori*; IL, interleukin; SD, standard deviation.

The CD4^+^ T cells express the FOXP3 transcription factor function as Treg cells, and Th22 T cells express the IL‐22. In this paper, the number of IL‐22^+^ CD4^+^ and Foxp3^+^ CD4^+^ T cells was measured by flow cytometry assay (Figure 3). It indicated that in peripheral blood of patients with *H*. *pylori* infection, the percentages of the IL‐22^+^ CD4^+^ and Foxp3^+^ CD4^+^ T cells were raised notably (*p* < .01).

### 3.5 Correlation analysis between the levels of IL‐22 and Foxp3 mRNA and the degree of *H. pylori* colonization and gastric mucositis

**Figure 4:**
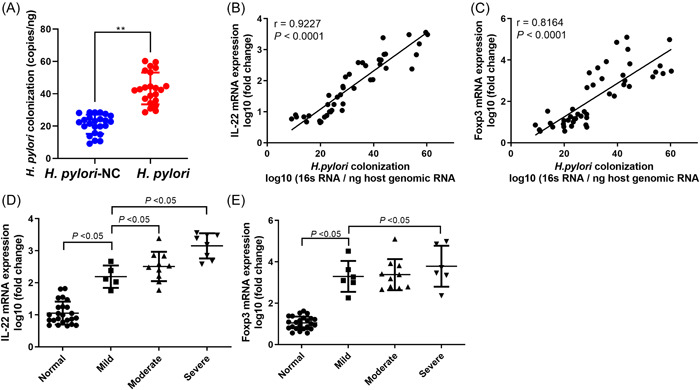
Correlation analysis. (A) The degree of *H. pylori* colonization in the *H. pylori*‐NC group and the *H. pylori* group. (B, C) The results of the Pearson's correlation analysis between IL‐22 and Foxp3 mRNA expression levels and the number of *H. pylori* colonization in the gastric mucosa of patients with chronic gastritis. (D, E) The results of the Pearson's correlation analysis between IL‐22 and Foxp3 mRNA expression level and the degree of gastric mucositis in the gastric mucosa of patients with chronic gastritis. Foxp3, forkhead box P3; *H. pylori*, *Helicobacter pylori*; IL, interleukin.

The degree of *H. pylori* colonization in the *H. pylori* group was markedly higher than that in the *H. pylori*‐NC group (*p* < .01, Figure 4A). And correlations between the levels of IL‐22 and Foxp3 mRNA and the degree of *H. pylori* colonization and gastric mucositis were assessed by Pearson's correlation analysis (Figure 4B−E). The levels of IL‐22 and Foxp3 mRNA were in direct proportion to the amount of *H. pylori* colonization (*r* = .9227 and *r* = .8164, *p* < .001). And their mRNA levels were also in direct proportion to the degree of gastric mucositis, respectively (*p* < .05). Additionally, the partial correlation analysis of IL‐22^+^ CD4^+^ T cells and Treg cells in peripheral blood of patients with *H*. *pylori* infection showed that after excluding the influence of IL‐22^+^ CD4^+^ T cells, the percentages of Foxp3^+^ Treg cells were significantly correlated with the *H. pylori* colonization, and the coefficient of partial correlation was 0.361 (*p* < .05). Furthermore, after excluding the influence of Foxp3^+^ Treg cells, IL‐22^+^ CD4^+^ T cells were significantly correlated with the *H. pylori* colonization, and the coefficient of partial correlation was .422 (*p* < .01).

## 4 DISCUSSION

*H. pylori* colonizes the gastric mucosa, and if it is not interfered with, the infection will last a lifetime.^22^ According to epidemiological data, *H. pylori* infection is transmitted through oral, fecal‐oral, or iatrogenic, especially water, which has a potential source of infection transmission.^23^ The infection is associated with the development of gastric complications, such as gastritis, gastric ulcer, and even gastric cancer.^24^ Modern research indicates that *H. pylori* can cause gastritis and gastric cancer.^25^, ^26^ Therefore, this paper studied the chronic gastritis patients infected with *H. pylori*, to provide a research basis for the mechanistic study of *H. pylori*‐related gastritis and the exploration of methods to eradicate *H. pylori*. In this experiment, 46 patients with chronic gastritis were recruited, 22 of whom were infected with *H. pylori*. There was no significant difference in their gender, age, and duration of illness. Also, there was no significant difference in the number of smoking and drinking between the negative and positive patients.

In serum, this study found the levels of gastritis‐associated cytokines, gastrin, PGI, PGII, and gastrin‐17, were significant differences. A study about gastrin reported that *H. pylori* led to hypersecretion of gastrin in serum which is due to stimulating antral G cells by inflammatory cytokines.^27^ Gastrin has also been proven could enhance growth factor secretion in rabbit gastric parietal cells in combination with IL‐1β.^28^ Additionally, scientists suggest that G‐17 is more suitable as a noninvasive biomarker of atrophic gastritis than total gastrin and it's the major form of the antral hormone gastrin in plasma or antral mucosal tissue, can regulate the secretion of gastric acid and the growth of gastric mucosal.^29^ A cross‐sectional study from the West China hospital demonstrated the value of G‐17 as a measurement marker for predicting atrophic gastritis in China.^30^ In addition, PGI and PGII can be used as biomarkers of chronic gastritis, but our study found no significant differences between patients with *H. pylori* gastritis and those with control patients.^31^ In short, the elevations of serum gastrin may have relevance to *H. pylori* infection and warrant further exploration.

In this study, we also found that *H. pylori*‐infected patients with chronic gastritis had more severe histopathological damage of gastric mucosa with significantly higher levels of inflammation‐related cytokines. The IL‐22, TGF‐β, TNF‐α, IL‐4, and IL‐17A levels were significantly upregulated in *H. pylori*‐infected patient blood. The ROC curve analysis indicated that for the differential diagnosis of *H. pylori* infection, above‐mentioned inflammation‐related cytokines levels are suitable. In addition, the decreased ratio of IL‐17A to IL‐22 indicated that increased serum levels of IL‐22 are not only related to Th17. Normally in mouse T cells, IL22 expression is thought to be closely associated with Th17.^32^ However, IL‐22 as a signature‐cytokines of human Th22 cells was increased in *H. pylori*‐infected patients.^33^, ^34^ In human CD4^+^ T cells, IL‐22 does not correlate with the expression of either IL‐17, of which Th22 cells are the major producer of IL‐22.^8^, ^34^, ^35^, ^36^ The above results combined with the flow cytometry results suggest that the increase of Th22 cells may be the key immune cells of chronic gastritis with *H. pylori* infection. Additionally, IL‐22 and IL‐17 work synergistically to induce antimicrobials and chemokines.^37^ IL‐17A has been proven that play a role in neutrophil recruitment to generate mucosal immunity to extracellular pathogens.^38^ Additionally, the epithelial cells activated by IL‐22 and IL‐17A can inhibit the growth of *H. pylori* in vitro, however, *H. pylori* may evade signaling by inflammatory factors such as IL‐22 by depletion of cholesterol,^39^ and the upregulation of IL‐22 may be due to its downstream signaling being blocked. The immune cells with IL‐22 secretion‐related deserve further investigation.

Furthermore, TGF‐β, which was raised in serum of patients with *H. pylori* infection, which is a powerful anti‐inflammatory cytokine, induced and maintain Treg cells.^40^ While, Nguyen with coworker reported that the *H. pylori‐related* protein cytotoxin‐associated gene A (CagA), the gene plays major pathogenic action, and it was a key protein in the inhibition of the TGF‐β signaling pathway,^41^ suggesting blocking effect of *H. pylori* on TGF‐β signaling pathway. Besides, this study proved that IL22^+^ CD4^+^ T cells and Foxp3^+^ Treg cells were positively correlated with *H. pylori* colonization and the severity of chronic gastritis. In recent years, researchers reported that Foxp3^+^ Treg cells attenuate immune responses partly via TGF‐β or IL‐10.^42^ Interestingly, scientific research has found that TGF‐β and IL‐6 regulated the differentiation of Th17, Th22, and iTreg cells.^43^ It can be seen that TGF‐β has a complex regulatory mechanism in *H. pylori*‐infected gastritis, and it may relate to the level of IL22^+^ CD4^+^ T cells and Foxp3^+^ Treg cells. Moreover, this study performed partial correlation analysis to judge internal relevance of IL22^+^ CD4^+^ T cells, Foxp3^+^ Treg cells, and *H. pylori* colonization suggesting that after correcting, IL‐22+ CD4+ T cells and *H. pylori* colonization are more relevant. Previous studies reported that there was a correlation between Th22 cell number and *H. pylori* density.^33^ However, influence of Th22 cells on this correlation is uncertain. This study suggests that Treg cells and IL‐22+ CD4+ T cells may independently respond to *H. pylori* infection.

Furthermore, a study proved that IL‐22 could enhance TNF‐α‐induced CXCL8 secretion to aggravate inflammation.^44^ In addition, INF‐γ, IL‐4, and IL‐17A, which are secreted by Th1, Th2, and Th17, were raised in *H. pylori* infection patients suggesting most immune cells are activated to participate in the inflammatory response. Among them, IL‐4 has been proven to be involved in the effect of gastric epithelial cells on the regulatory tolerance and immune response balance.^45^ In conclusion, Treg and Th22 cells may be involved in the development of *H. pylori*‐induced chronic gastritis.

As for the limitation of this study, first, IL‐22, IL‐17A, and other cytokines usually involve different immune cells, and more kinds of T cells are worth exploring. For example, a study has reported that IL‐22 is produced in Th22, Th17, Th1 cells, innate lymphoid cells, and some non‐lymphocytes,^46^ although, in human T cells, Th22 cells are the predominant cells expressing IL‐22,^32^ it's still necessary that more definitive discrimination of the cell types within the IL22^+^ CD4^+^ cells. In addition, the deeper biological mechanism of Treg and Th22 cells is worth further exploring, and the mechanism of action between the immune cytokines of Treg and Th22 cells and *H. pylori* infection remains to be more thoroughly investigated. We will study the biological mechanism of *H. pylori* on Th22 and Treg cells based on sufficient conditions in the future.

## 5 CONCLUSION

This study reported that there are differences in the level of inflammation‐ and gastritis‐related indicators between *H. pylori*‐positive and negative chronic gastritis patients. The ROC curve analysis indicates that the levels of IL‐22, TGF‐β, TNF‐α, IL‐4, IL‐17A, and IFN‐γ in serum are suitable for differential diagnosis of *H. pylori* infection. Through H&E staining, ELISA, qRT‐PCR, and flow cytometry analysis, it was demonstrated that patients with *H. pylori* infection have more severe tissue damage and higher inflammation levels in gastric mucosal and serum, and proved that number of Treg and Th22 cells were raised in *H. pylori*‐infected patients, and the mRNA levels of their marker gene IL‐22 and Foxp3 were closely related with the degree of *H. pylori* infection and the severity of gastritis. This study provides an experimental basis for the study of eradication of *H. pylori* and the biological mechanism of chronic gastritis.

## AUTHOR CONTRIBUTIONS

Biyu Yao, Zhiming Huang concept and designed this study. Biyu Yao, Xiaoyan Xu, Weijie Liu acquisited data. Biyu Yao, Qin Zhang, Wei Wang analyzed and interpretated data. Biyu Yao, Xiaoyan Xu, Weijie Liu, Qin Zhang, Wei Wang drafted tha manuscript. Zhiming Huang revised the manuscript. Biyu Yao obtained funding support. All authors agree to submit and publish this article.

## CONFLICT OF INTEREST

The authors declare no conflict of interest.

## ETHIC STATEMENT

The study protocol was approved by the Ethics Committee of the People's Hospital of Yuhuan (Zhejiang, China).

## Supporting information

Supporting information.

## Data Availability

The data that support the findings of this study are available from the corresponding author upon reasonable request.
