# Supplementary figures and images for: The correlation of Th22 and regulatory T cells with Helicobacter pylori infection in patients with chronic gastritis
Source: Immun Inflamm Dis. 2023 Jan 18;11(1):e768. doi: 10.1002/iid3.768 (PMC9846114; doi:10.1002/iid3.768)

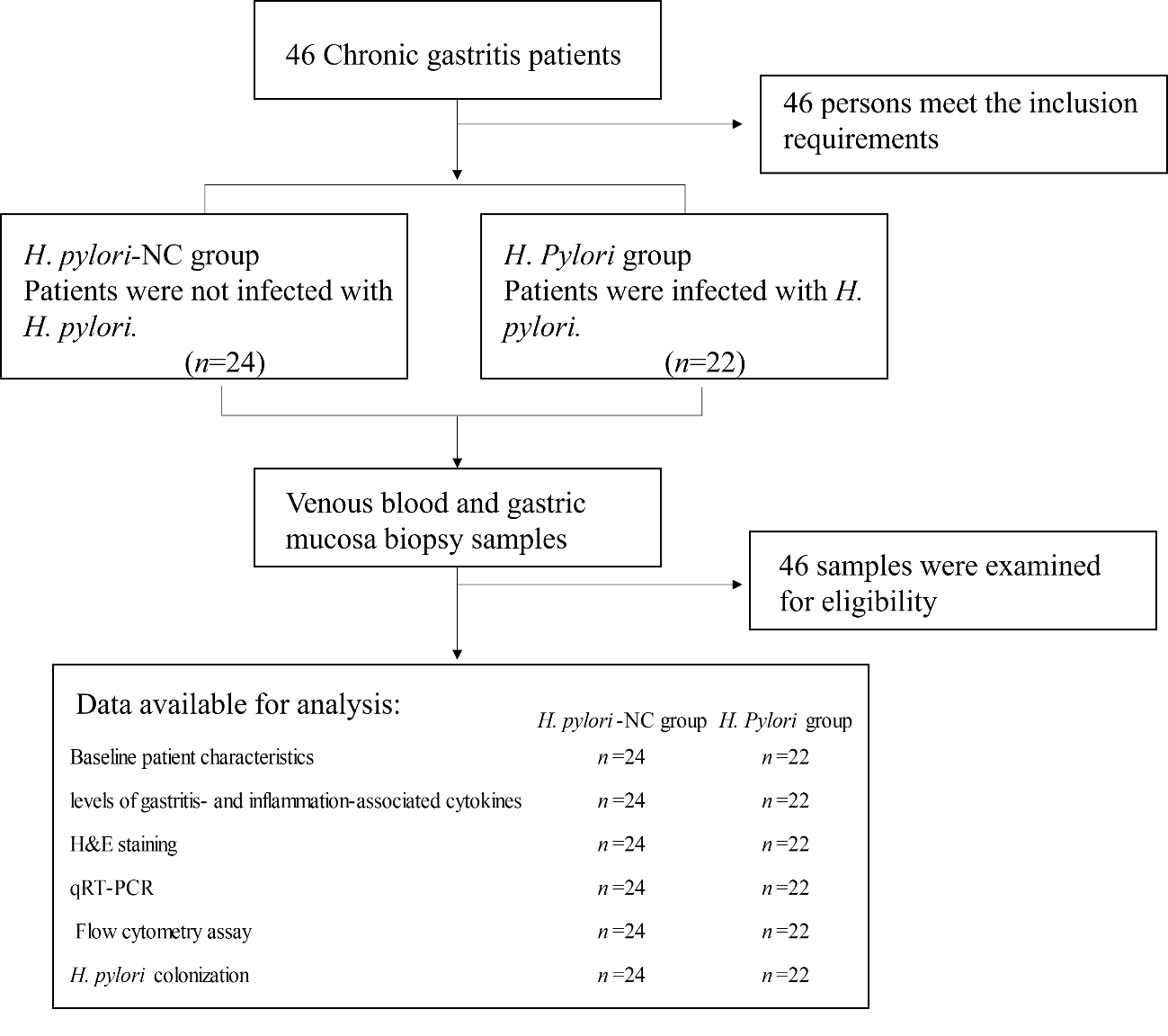


**The Supplementary Figure 1 The flow diagram of experimental process.**

Supplement: Supplementary file 1 — Supporting information. [file IID3-11-e768-s001.docx]
